# Integrating fire safety into bridge design is essential for resilient infrastructure

**DOI:** 10.1038/s41467-024-49593-3

**Published:** 2024-08-05

**Authors:** Andrea Franchini, Bosibori Barake, Carmine Galasso, Maria E. Moreyra Garlock, Joseph Mulligan, Spencer Quiel, Jose L. Torero

**Affiliations:** 1 Dept. of Civil, Environmental and Geomatic Engineering, University College London, London, UK; 2 Kounkuey Design Initiative, Nairobi, Kenya; 3 Dept. of Civil and Environmental Engineering, Princeton University, Princeton, NJ USA; 4 Dept. of Sustainable Development, Environmental Science and Engineering, KTH Royal Institute of Technology, Stockholm, Sweden; 5 Dept. of Civil and Environmental Engineering, Lehigh University, Bethlehem, PA USA

**Keywords:** Civil engineering, Civil engineering, Decision making, Developing world

## Abstract

The frequent occurrences of bridge fires and the substantial disruptions and direct/indirect economic losses resulting from these events highlight the immediate need for effective fire-safety-oriented design of new bridges and retrofit approaches for vulnerable existing bridges. In this Perspective, we discuss why a holistic engineering approach integrating innovative fire analysis methods and structural design/retrofit strategies into multi-hazard and future-oriented risk modeling frameworks represents the way forward to more sustainable and resilient infrastructure in an uncertain and rapidly changing built environment.

## Abstract (web summary)

Bridge fires cause significant disruptions and economic losses in modern society, yet fire hazards are still often ignored or oversimplified in bridge design. This Perspective emphasizes the need for more holistic and comprehensive fire-safety design when retrofitting or designing new bridges.

## Introduction

**Fig. 1:**
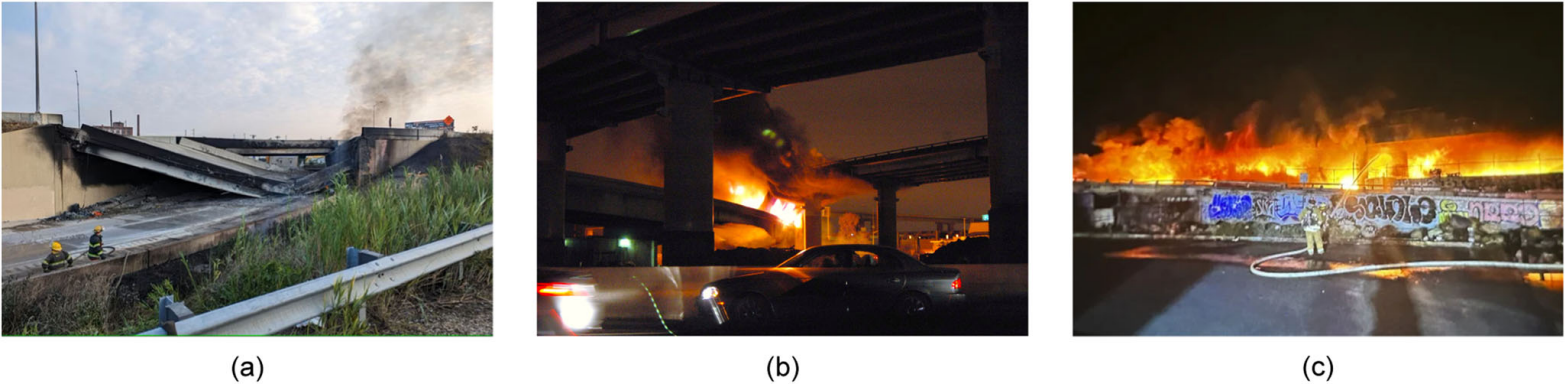
Examples of bridge fires. **a** Collapsed I-95 overpass in Philadelphia, Pennsylvania, USA, following a fire on June 11, 2023 (Photo credit: Philadelphia Office of Emergency Management). **b** MacArthur Maze bridge fire in Oakland, California, USA, on April 29, 2007 (Photo by Philip Liborio Gangi). **c** I–10 bridge fire in Los Angeles, California, USA, on November 11, 2023 (Copyright 2023 California Department of Transportation, all rights reserved).

In the early morning of June 11, 2023, flames and smoke plumes engulfed both sides of the I-95 overpass in Philadelphia, Pennsylvania, USA. Videos captured drivers experiencing jolts as their cars crossed a falling section of the road, witnessing the gradual collapse of the bridge girders. The highway was shut down within minutes, and the bridge collapsed 25 minutes after the fire was reported (see Fig. 1a; e.g., refs. ^1,2^). The fire was caused by a fuel tanker truck’s collision with a bridge abutment. Despite the partial reopening of six lanes on June 23, 2023, several months of repair were still needed (e.g., refs. ^2,3^). *Was a similar fire scenario—and its potential consequences—considered when defining the bridge design?*

**Fig. 2:**
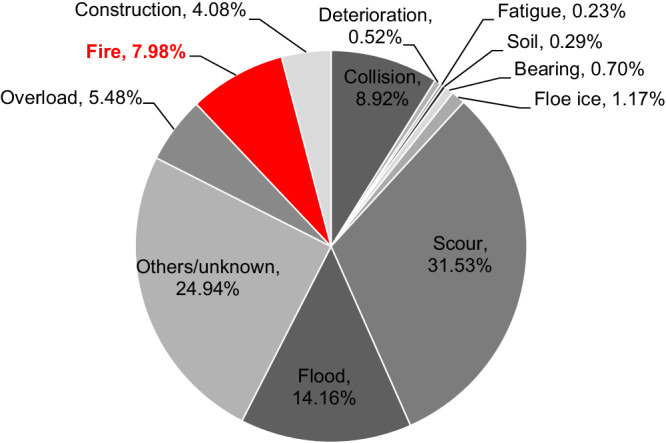
Cause of surveyed bridge failures in the period 1807–2021^12^.

The collapse of the I-95 bridge, which carries about 160,000 vehicles daily^2^, is just a recent example of a long series of bridge fires causing significant disruptions and economic losses over the past two decades and impacting large populations (e.g., refs. ^4–6^). For instance, the collapse of the MacArthur Maze I-80/880 interchange in Oakland, California, USA, in 2007 (see Fig. 1b) resulted in repair costs of $9 million and an economic impact (in terms of repair cost, traffic management, and business interruption) of approximately $156 million^4,7^; the rebuilding of the Mathilde Bridge in Rouen, France, in 2012, implied direct and indirect costs of about $8.6 and $10.7 million, respectively^8^; emergency funding of $12 million was allocated to manage the traffic and repair the collapsed section of the I-75 Brent Spence Bridge in Kentucky, USA (2020), with reconstruction costs reaching $3.1 million^9^. Peris-Sayol et al.^10^ examined 154 bridge fires to identify the main factors determining damage. Lee et al.^11^ compiled data on 1254 bridge failures from 1980 to 2012, revealing that 2.13% were due to earthquakes, 1.81% to wind, and 3.20% to fires. This latter hazard accounted for 7.98% of the 1716 bridge failure events collected by Xiong et al.^12^, representing the 1807–2021 timeframe (see Fig. 2). Another report^13^ concluded that one bridge per year in the USA is expected to permanently lose service because of fire. As of November 11, 2023, while we write this article, another fire incident has halted traffic on the I-10 in downtown Los Angeles, California (Fig. 1c), one of the busiest routes in the USA^14^.

Despite the evident threat that bridge fires pose to society, fire hazard is often treated in an oversimplified manner in structural analysis and design. The unique and complex interactions between bridges and fires are poorly understood, and conventional design approaches incorporate fire as an add-on to design solutions optimized for many other ordinary and extreme loads. Explicit fire performance criteria associated with bridges’ structural design receive limited attention in design codes and standards. For instance, NFPA 502^15^ (which offers some of the few available standardized guidelines for bridge fire design) requires designing structural elements to (i) support firefighter accessibility; (ii) minimize economic impact; and (iii) mitigate structural damage. However, how to quantify such criteria is not explained. Moreover, these provisions do not apply to bridges with spans shorter than 300 m, despite most bridges damaged by fires falling within this category (e.g., ref. ^10^). A designer is given little guidance on determining “design fire scenarios” based on traffic data and implementing fire protection measures. Several reasons contribute to such a limited consideration of fire hazards in bridge design:

- Fires of a magnitude that can affect the integrity of bridges are considered rare events (despite their frequent occurrence). As a result, public perception tends to underestimate the actual cost of these accidents (e.g., ref. ^5^).
- There is a higher occurrence of building fires as opposed to bridge fires (e.g., ref. ^16^). For example, Peris-Sayol et al.^10^ identified 111 bridge fire incidents in the United States from 1997 to 2015. During the same period, Ahrens and Maheshwari^17^ report 7,192,500 home fires.
- Lower uncertainties are associated with defining fire scenarios for compartment fires (i.e., fires that develop in an enclosure or “compartment” within a building^18^), where flashover can be assumed, and the heat fluxes can be determined from compartment properties and content (e.g., ref. ^16^).
- Life safety is the primary objective of fire-safe design. In this sense, large open spaces in bridges allow people to be evacuated fast enough in case of fire, and therefore, casualties can be generally avoided (e.g., ref. ^19^).
- The media’s tendency to spotlight building fires that cause significant casualties and the fact that large populations generally do not congregate around bridges amplify the perception of building fires being more relevant than bridge fires.

Nevertheless, in a world witnessing rapid urban population growth with cities expanding and/or densifying at unprecedented rates, ensuring safer and more resilient infrastructure (e.g., ref. ^20^), including transport infrastructure and its components (e.g., bridges), becomes crucial for sustainable urban development. Within this context, the lessons learned and knowledge derived from the described events, coupled with the lack of fire-safety-oriented design approaches for bridges, create an opportunity to *Design Better* future infrastructures and *Build Back Better*^21^ damaged ones. *But how can these goals be achieved?*

**Fig. 3:**
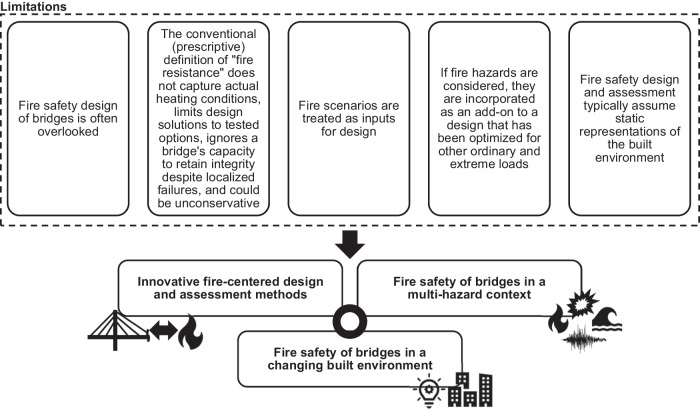
Identified limitations in the fire safety design of bridges and organization of the manuscript.

We argue that implementing a holistic engineering approach integrating adequate design and analysis methods for all relevant hazards, including fire, is necessary and can address the abovementioned challenges. This approach comprises two elements: (i) using innovative, fire-centered design and assessment methods aimed at controlling fire dynamics and providing explicit measures of performance (see section “Innovative fire fire-centered design and assessment methods”); and (ii) integrating such novel methods into multi-objective and multi-hazard design frameworks (see section “Fire safety of bridges in a multi-hazard context”). These frameworks should leverage future-oriented risk perspectives that enable capturing the impact of fire safety on a dynamically changing built environment and vice versa (see section “Fire safety of bridges in a changing built environment”). Figure 3 summarizes the manuscript’s organization and the limitations we identified in the current practice. Furthermore, at the end of the paper, Table 1 provides an overview of our recommended actions.

## Innovative fire-centered design and assessment methods

### Changing perspective: fire scenarios as outputs

Bridge fire safety reviews by Garlock et al.^4^, Nicoletta et al.^22^, Hu et al.^5^, and Liu et al.^6^ (among others) show that existing studies have predominantly focused on improving the fire performance of specific bridge typologies (involving different structural systems, such as beam, arch, and cable-supported bridges, and different materials, such as steel, concrete, and steel-concrete). In contrast, we aim to present a technology-agnostic analysis of existing performance quantification and optimization methods for designing new bridges or assessing, retrofitting, and protecting existing ones.

Structural resistance to ordinary (e.g., gravity, traffic) and extreme (e.g., earthquake, hurricane) loads is calculated using models that attempt to capture/mimic actual loading conditions. Differently, “fire resistance” is traditionally defined as the time for which a structural element or component continues to perform its function during a standard temperature history imposed by a furnace (e.g., ref. ^23^). Standard fire curves are also frequently used to assess structural performance through numerical analysis (e.g., refs. ^24–26^). While all standards and guidelines globally use the same temperature histories, the definition of “performing its function” varies, with the most common failure criteria being the attainment of a critical temperature (directly associated with loss of material strength) on a specified element or location.

As a result, this approach has several limitations. First, its prescriptive nature implies limiting the spectrum of design solutions to tested options, hindering innovation and cost-effectiveness (e.g., ref. ^27^). Furthermore, the heating regime in a real fire is significantly different from that of the test (e.g., ref. ^18^). Frequently, performance is assessed only from a thermal perspective (i.e., without any structural analysis) and using thermal exposure conditions that are insufficient and sometimes inappropriate (e.g., ref. ^28^). Especially relevant is the fact that a structure’s capacity to retain integrity despite localized or component failure is neglected. In addition, heat-induced forces and relative temperature deformations between structural elements (rather than localized element failure) often determine structural collapse (e.g., refs. ^29–31^). Thus, extrapolation of “fire resistance” testing results does not provide an explicit quantification of the performance of a structure when exposed to a real fire (e.g., refs. ^32,33^).

To cope with these limitations, the possibility of calculating structural fire performance by applying more rational engineering methods has emerged in the context of performance-based fire design. Performance-based design involves demonstrating structural performance rather than adhering to prescriptive regulations (e.g., refs. ^23,34^). Through this practice, innovative and non-conventional design solutions (e.g., new forms and new materials) can be used to comply with performance objectives (e.g., ref. ^35^). Critical to demonstrating structural performance is consideration of *fire-structure coupling effects*. Indeed, fire is a phenomenon that evolves spatially and temporally as a function of the structure it develops within and interacts with. Similarly, the heating regime of the structure develops in response to the fire (e.g., refs. ^36–38^).

The performance-based design also demands addressing a multitude of epistemic and aleatory uncertainties that influence the design process^39^. A way to address these uncertainties is through probabilistic methodologies (e.g., fragility analysis and/or risk *assessments*^40,41^) that can aid in prioritizing vulnerable infrastructure sections for retrofitting (e.g., ref. ^42^). Franchini et al. ^43^ provide a broad discussion on the benefits and limitations of conventional probabilistic performance-based analysis methods for *design* purposes.

A review of the work discussed above shows that for most studies where advanced structural analyses were conducted (e.g., refs. ^24–26,44,45^), the applied thermal conditions were generally oversimplified or inaccurate (e.g., standard and hydrocarbon temperature curves that impose heating conditions akin to the furnace test^36^). Earlier studies have demonstrated that using inaccurate thermal and mechanical boundary conditions leads to inaccurate estimation of the fire performance of bridges^46^. Other approaches consider more relevant thermal exposures but do not include structural analysis, resulting in similar inaccuracies (e.g., ref. ^47^). In an attempt to improve on these studies, advanced computational fluid dynamics (CFD) and finite element models (e.g., refs. ^46,48,49^) have been coupled, showing the value of describing fire and structure coupling effects. Nonetheless, these studies also show the complexity of these simulations and their extreme computational cost. As a result, the practical value of these precise calculations is often limited, especially at the design stage, potentially favoring the combination of intermediate fire and heat transfer models (e.g., refs. ^40,47,50,51^) with finite element analysis of structural performance.

The different time scales associated with fire growth and structural heating provide a simpler alternative that can still capture the fire-structure coupling effects, enabling approaching them almost independently. While this simplification has a lengthy history, it has predominantly adopted a worst-case scenario approach in defining fire characteristics. This has resulted in the continuous enhancement of structural strength. Yet, when fire-structure coupling effects exist, and the fire affects a structure as much as the structure alters fire behavior, the structural design can control and positively affect fire scenarios.

**Fig. 4:**
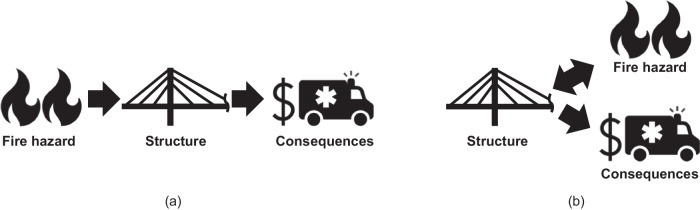
Fire safety assessment and design methods. **a** Conventional approach that considers fire hazard scenarios as analysis inputs. **b** Perspective shift, which treats fire scenarios as output design variables to optimize.

In other words, the structural features chosen during the design phase determine the fire intensity (i.e., its damage potential, for instance, in terms of the temperature field that forms across structural components). This reveals a designer’s ability to treat (and control) fire scenarios as design variables, signifying a fundamental shift in viewpoint on the fire safety design of structures. Indeed, regardless of the modeling accuracy, fire scenarios are traditionally treated as analysis inputs. Then, the structural response is computed, and consequences are estimated (Fig. 4a). Conversely, considering these scenarios as design variables means they become analysis outputs (Fig. 4b). From a performance quantification (i.e., assessment) perspective, the fire-structure coupling effect means that fire scenarios maximize consequences are structure-specific. Consequently, assumptions inherent in treating fire scenarios as analysis inputs may not capture the full extent of maximum consequences.

The fire-structure coupling effect should not necessarily be resolved in detail to achieve an adequate and precise analysis. Rather, it can be exploited to optimize the design. In this sense, the fire hazard is created by how the bridge is designed, and an appropriate design can reduce maximum consequences to a desired level. A few examples of such design decisions are presented below:

- Barriers on the deck, cable locations (for cable-supported bridges), and under-deck clearance, influencing possible locations where a fire might start.
- The deck shape and materials, affecting heat feedback to the fuel bed, flame spread, and heat concentration (e.g., if stiffeners, ribs, or bracings confine the ceiling jet flow).
- The layout and number of lanes, impacting fire spread during traffic congestion and response time for fire services.
- The deck pavement slope (longitudinal and transverse) and the water management system, influencing fire development above and below the deck by allowing for pooling of liquid/molten combustible materials.
- The presence of flammable construction materials, including wooden components or those used for cable environmental protection (in cable-supported bridges), potentially contributing to further fire spread.

Recently, Franchini et al.^38^ introduced a fire safety design methodology named the Consequence-oriented Fire intensity Optimization (CFO) approach that (i) treats fire scenarios as additional design variables; (ii) optimizes the balance between increasing structural capacity and decreasing fire intensity to bound maximum fire consequences within a selected threshold; (iii) incorporates uncertainty propagation techniques to assess the impact of chosen uncertainty sources on the estimated consequence metric(s). Features (i) and (ii) contrast with the current practice that tends to *assess* the fire performance of a structure designed for conventional and extreme loads other than fire and then, if required, designs active and/or passive protection measures (e.g., refs. ^24,52–54^). Feature (iii) facilitates future design strategies that enhance robustness to uncertainty, including deep uncertainties (e.g., ref. ^55^). The CFO approach has been further developed to optimize the inherent fire capacity of structures, defined as their ability to retain integrity/functionality without additional fire safety measures^56^. The inherent capacity is a crucial parameter for bridge fire safety because implementing active and passive fire protection measures for these structures may be costly, require significant maintenance, or be impractical (e.g., ref. ^5^).

From an assessment perspective, the CFO approach can, be utilized to identify structure-specific features leading to fire scenarios that maximize consequences. Similar outcomes can be achieved through the Maximum Allowable Damage approach^57^, which updates fire scenario assumptions during the analysis to identify the “maximum damage potential.”

An alternative approach is to employ inverse fire analysis techniques (e.g., ref. ^58^), which begin with a selected temperature field across components, identify the fire scenario features causing it, and assess whether such scenarios are physically plausible^58^. This approach can be computationally costly and suffers from the complexities and non-linearities of the physical phenomena involved as well as from compensation effects, leading to significant problems associated with establishing the uniqueness of the solution.

### Applicability contexts: design, assessment, and retrofit

Several technical aspects discussed above require different considerations based on whether one is designing a new bridge, assessing an existing structure, investigating a retrofit solution, conducting a post-fire assessment, or performing a forensic analysis. While we focus on the former contexts in this perspective, readers interested in the latter two are encouraged to explore relevant literature (e.g., refs. ^4,30,46,59^) for initial insights.

A new design enables more flexibility in decisions aimed at diminishing fire intensity. In this context, a process that incorporates verification of performance against a set of simplified thermal conditions could still achieve desired performance objectives (e.g., Law and Bisby^33^ discuss that an element performing better in a furnace test is generally expected to perform better in the case of a fire). Nevertheless, the simplicity of fire and heat transfer models defines how much a designer can leverage the fire-structure coupling effect to obtain more optimized (e.g., more cost-effective, more sustainable) solutions^38^.

For retrofit solutions, many (if not all) of the variables that affect fire intensity might be unavailable to a designer (e.g., bridge clearance, girder geometry). Yet, considering fire scenarios as analysis outputs facilitates designing the retrofit system for the conditions that maximize consequences. Similarly, the discussed methods allow analyses in the low-probability high-consequence region of the consequence spectrum for assessing existing bridges against potential fire events.

In the considered methods, the uncertainty treatment approach differs depending on whether one considers a new design or a retrofit/existing bridge assessment. Specifically, aleatory and epistemic uncertainty effects evolve over a bridge’s life cycle, with the potential of reducing the latter by means of inspection and monitoring (e.g., refs. ^60,61^). Cadena et al.^62^ provide an overview of uncertainty-based decision-making methods for fire safety engineering. Here, we advocate for analysts to be explicit about the uncertainty effects they aim to assess and the calculation method they select for that purpose.

### Performance objectives

While current structural fire engineering primarily addresses life safety performance objectives (traditionally, the main focus for buildings), we believe innovative fire-centered design and assessment methods should also explicitly consider a spectrum of damage states (e.g., superficial, moderate, heavy, and hazardous damage^40^). This enables design decisions aimed at limiting repair costs or achieving post-fire resilience and rapid functional recovery (i.e., low downtime). The ability to reopen a bridge to traffic rapidly after a fire event can be critical, as observed, for instance, in the recent fire events in Philadelphia^1^ and Los Angeles^14^.

In our view, considering factors such as bridge downtime or repair costs as explicit performance objectives would have several implications:

- Technical implications. Distinct and potentially conflicting design strategies may be required depending on the target performance objective. For example, minimizing the post-fire functional recovery time could require most of the structure to remain elastic or the use of rapidly-replaceable elements, irrespective of their cost. Conversely, the repair cost may be reduced by allowing inelastic mechanisms for some structural elements, regardless of their repair/replacement time. Meanwhile, solutions favoring both objectives could exist. For instance, fire-exposed steel members that are “noticeably deformed” but remained below the phase change temperature could be heat straightened if this choice is economically justified^63^. As a result, professional competency (e.g., ref. ^64^), is essential to conceive and navigate through the array of potential design solutions.
- Economic implications. While incorporating fire safety measures to control downtime or repair costs might increase the initial construction costs, it could save money over the structure life cycle. Additionally, properly designed bridges could attract lower insurance premiums, further saving costs. Prioritization and classification of new and existing bridges for intervention within an infrastructure network is required to cope with the limited availability of funding for public infrastructure (e.g., refs. ^42,65^).
- Political implications. Legislatures and government agencies should develop policies to facilitate repair cost- and/or functional recovery-based fire design. These policies require identifying acceptable repair costs and downtime through community-level cost-benefit analysis and community engagement. A broader discussion of these concepts (for seismic design) can be found in a white paper by the Earthquake Engineering Research Institute (EERI)^66^.

### The role of modern technologies

The innovative fire-centered design and assessment methods discussed herein necessitate utilizing traditional fire analysis tools (e.g., finite element analysis, CFD) to capture and better understand fire-structure coupling effects. Meanwhile, modern technologies such as Artificial Intelligence (AI) and Machine Learning (ML) are increasingly permeating structural engineering practices (e.g., refs. ^67,68^). In the context of this work, we foresee these modern technologies will complement the fire safety of bridges in several ways:

- Surrogate modeling (e.g., ref. ^69^) can enhance computational efficiency in optimization, uncertainty quantification (e.g., ref. ^70^), and, more generally, in the iterative process required to treat fire scenarios as output (design/assessment) variables.
- AI and ML algorithms can help understand and better model complex patterns and relationships (e.g., ref. ^71^) among the factors influencing fire-structure coupling effects.
- Treating fire scenarios as design variables uncovers a whole set of design solutions aimed at controlling fire intensity; thus, leveraging ML for design space exploration could unveil innovative solutions beyond human intuition and creativity (e.g., reinforcement learning algorithms enable exploiting machine “creativity,” generating novel designs from the combination of familiar solutions^72^);
- In the context of multi-hazard design and life cycle analysis (discussed in the next sections), structural health monitoring systems allow for reducing uncertainty in risk and resilience analysis^60,73^. AI and ML enable faster data interpretation and enhanced damage detection (e.g., refs. ^74,75^).

## Fire safety of bridges in a multi-hazard context

Innovative fire safety design approaches aimed at controlling fire dynamics to reduce fire intensity (as the CFO approach) find optimal use within a holistic design practice. This involves considering fire safety from the early design stages, enabling the optimization of many design variables. A holistic design approach emerges as the key to achieving truly optimized structures (e.g., refs. ^37,76^), and efforts should be directed towards adopting such a strategy.

**Fig. 5:**
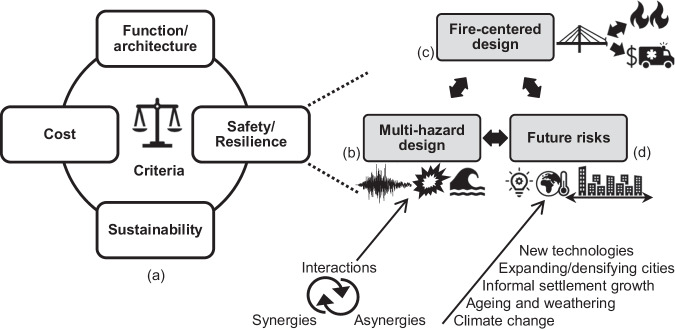
Recommended holistic design approach for fire safety of bridges in a multi-hazard context and a changing built environment. **a** Multiple criteria. **b** Factors involved in multi-hazard design. **c** Fire-centered approach, accounting for the unique features of the fire phenomenon and its interaction with the considered infrastructure. See also Fig. 4. **d** Factors affecting future fire risk in a changing built environment.

The term “holistic” implies a “multicriteria” approach, encompassing function/architecture, cost, sustainability, and safety/resilience (see Fig. 5a). The considered criteria can be weighted differently according to the involved stakeholders and their preferences, making the design also “user-centric.” “Safety” addresses both ordinary and extreme loading conditions. A structure may face multiple (often interacting) hazard events throughout its lifecycle. Hence, multi-hazard design strategies (e.g., refs. ^77,78^) are essential in a holistic design approach. In this regard, the *Sendai Framework for Disaster Risk Reduction* underscores the importance of multi-hazard and multi-sectoral risk reduction practices^21^.

The multi-hazard design represents an innovative approach in structural engineering, aiming to enhance the performance of structural components and systems by holistically addressing the various hazards such components and systems will face during their lifetime (e.g., ref. ^79^). The key distinction between the traditional “design for multiple hazards” as outlined by design codes and a “multi-hazard design philosophy” lies in the latter’s direct consideration of interactions, synergies, and asynergies among hazards (see Fig. 5b). In contrast, the conventional approach in design codes involves load combinations and load factors to account for multiple hazards, which are considered independent (e.g., refs. ^77,78^). However, due to interactions, the consequences entailed by multiple hazards might not be captured adequately through simple superposition of single-hazard effects (e.g., ref. ^77^).

Hazard interactions can be classified as occurrence (Level I) and consequence (Level II) interactions (e.g., refs. ^77,80,81^). Level I interactions refer to interdependencies in the hazard nature, distinguishing “non-interacting,” “concurrent” (i.e., hazards that tend to occur together), and “successive” (i.e., a primary hazard triggers or alters the frequency of a secondary hazard) events. The literature defines Level I interactions as “the natural interactions of hazards through actions produced at a given location (i.e., site effects) that are independent of the presence of physical components.” However, this definition is unsuitable for fire because, as discussed above, physical assets/systems create and define this hazard. Despite this inconsistency, classifying multi-hazard interactions based on site-specific effects seems appropriate. For example, a fire could be considered a blast’s (or an earthquake’s) site effect in a fire-following-blast (or fire-following-earthquake) scenario. Thus, we define site effects simply as “the natural interactions of hazards through actions produced at a given location.” Future studies should address this inconsistency, defining appropriate interaction classes that capture the nature of the fire hazard. Specifically, taking a bridge pier as an example, a fire following a blast-induced by a vehicle impact represents a triggering interaction (e.g., ref. ^82^). Differently, fire and earthquake could be considered non-interacting hazards for bridges: an earthquake would rarely trigger a bridge fire, whereas a negligible probability characterizes the simultaneous occurrence of an earthquake during a vehicle-induced fire. This fact is supported by Petrini et al.^83^ who observed that concurrent- or successive-type interactions between natural and human-induced hazards are rare for bridges. Yet, earthquake and fire can exhibit Level II interactions.

Level II interactions (e.g., ref. ^81^) encompass structural performance impairment through “shock deterioration” (i.e., damage caused by hazard events occurring at a specific point in time) and “gradual deterioration” (i.e., damage induced by aging and deteriorating mechanisms). As an example of shock deterioration, an earthquake could set the bridge in a minor damage state, increasing the vulnerability to a future fire. Similarly, fire-induced residual deformations or spalling could increase seismic vulnerability. We are not aware of studies addressing these phenomena for bridges—a gap that should be addressed soon; however, several studies on post-fire seismic performance (e.g., refs. ^84,85^) and post-earthquake fire performance (e.g., refs. ^86,87^) that address buildings can be found in the literature.

Gradual deterioration-type interactions also impact the fire safety of bridges. Indeed, while typically designed and assessed assuming pristine conditions, these structures undergo constant aging and environmentally-induced deterioration mechanisms (e.g., corrosion of steel girders and reinforcing steel rebars, cracking and spalling of concrete^88,89^). Over time, these effects can diminish bridges’ inherent fire capacity^56^. Similar impacts can be observed in passive fire protection measures (mainly cementitious and intumescent coatings). For example, weathering significantly reduces the protective performance of intumescent coatings (e.g., refs. ^90,91^). Regarding cementitious coatings, experiments indicate limited deterioration in fire resistance (determined through furnace and jet fire testing) over a decade^92^. However, corrosion was observed beneath certain coatings. In addition, coating physical damage (e.g., nicks, cracks, disbondment) reduces fire resistance (e.g., ref. ^93^).

With this background, a multi-hazard *design* approach (see Fig. 5b) must capture multi-hazard interactions and acknowledge that engineering structures and systems possess an inherent multi-hazard resilience (e.g., ref. ^94^), providing an opportunity for safer and more cost-effective design solutions. In this Perspective, the term “resilience” denotes the ability of a system to resist, respond to, and recover quickly from external shocks or perturbations over a defined period (e.g., refs. ^95,96^). The inherent resilience stems from cross-hazard synergies (e.g., refs. ^78,97^). As an illustration, increasing a bridge column height (and therefore the bridge clearance) reduces hurricane effects (e.g., deck unseating^98^) and fire effects (e.g., heat fluxes from a vehicle fire beneath the deck^38,99^).

Asynergies (i.e., opposing or conflicting effects) also exist (e.g., ref. ^100^), so it is crucial to consider that design measures targeting a specific hazard can potentially compromise structural performance under another hazard. In other words, asynergies create design trade-offs. Building upon the earlier illustration, increasing the bridge column height to improve hurricane and fire performance may result in higher seismic vulnerability due to the complex interplay between the global dynamic response and the components’ capacities^98^. Close cable spacing and light girders enhance the seismic performance of cable-stayed bridges; however, both these design choices increase the probability of fire-induced damage^101^. Another example is provided by constraining the longitudinal deck displacement at the towers. While this choice represents an efficient approach to limit the longitudinal earthquake-induced deck vibration, it increases the axial load in the deck during a fire up to a level that could induce buckling^102^.

A multi-hazard perspective also contributes to the sustainable design of structures, with “sustainability” generally measured in terms of environmental, economic, and societal impacts (e.g., ref. ^103^). Indeed, while resilience goals could conflict with environmental sustainability at the material production and construction stages (e.g., in terms of material efficiency), the economic (e.g., monetary losses, repair costs), societal (e.g., activity disruption) and environmental (e.g., carbon dioxide emissions associated with element repair and/or substitution) impacts of hazards could outweigh early-stage sustainability goals (e.g., refs. ^104,105^). Along similar lines, Bocchini et al.^106^ studied common features between resilience and sustainability, concluding that the two aspects are complementary and should be considered holistically. Finally, we observe that leveraging cross-hazard synergies enables minimizing material quantities required to ensure structural performance to multiple hazards, thereby reducing the environmental impact of the considered bridge and contributing to sustainability.

While research on multi-hazard bridge design is expanding (see the review by Roy and Matsagar^97^, among others), to date, only a few studies consider fire, and even fewer focus on bridge fires, despite the above background and statistics. When fire is considered, the emphasis has been on its interaction with impact and/or blast loads (e.g., refs. ^82,83^). Therefore, there is an urgent need for progress toward natural and human-induced hazard consequence analysis for bridges and other infrastructure systems. For example, future studies should explore how fire-centered design and assessment strategies (Fig. 5c; e.g., the CFO approach) can be integrated into multi-hazard lifecycle consequence calculation frameworks developed for natural hazards (e.g., ref. ^81^). As detailed in the following section and discussed in ref. ^107^, all the mentioned criticalities should also account for the effects of a changing built environment.

## Fire safety of bridges in a changing built environment

Fire safety design and assessment analyses typically assume static representations of the built environment. However, cities, infrastructure systems, and structures are in a constant state of innovation, expansion, evolution, and deterioration. This dynamism alters physical/social hazard exposure and vulnerability^20^. Moreover, fires ignite, develop (potentially to an uncontrolled state), and decay based on their surrounding environment, shaping fire dynamics and influencing the combustion process. As a result, the nature of the fire phenomenon and the potential scenarios threatening bridges, as well as other engineered systems, evolve with the built environment. In addition, fire sources near bridges are increasing due to urban trends and traffic changes (e.g., ref. ^4^). These facts highlight the relevance and pressing need for understanding and properly managing future risks–see Fig. 5d.

For instance, the automotive sector is progressively transitioning towards more sustainable solutions like electric vehicles. However, an unintended consequence of this innovation is the growing threat of battery fires (e.g., ref. ^108^), which can give rise to new and unforeseen fire scenarios. Another example is found in pultruded glass fiber reinforced polymer (GFRP) bridge decks, which are increasingly popular for retrofit (e.g., ref. ^109^) due to their lightweight, corrosion resistance, fast installation, high strength, and low lifecycle cost (e.g., ref. ^110^). These decks, when exposed to heat fluxes exceeding around 25 kWm^−2^, can quickly ignite and sustain flaming combustion (e.g., ref. ^111^), completely changing the foreseen fire scenario from a vehicle fire. As a reference, experimental heat fluxes from small vehicles range between 20 and 70 kWm^−2^ ^112^. In the future, 3D-printed GFRP bridges will enable low-cost, rapidly constructed, complex, and unique shapes (e.g., ref. ^109^). While this technology may reveal unexpected flame propagation paths and fire scenarios, it also presents opportunities for optimized solutions that minimize flame propagation and fire effects.

**Fig. 6:**
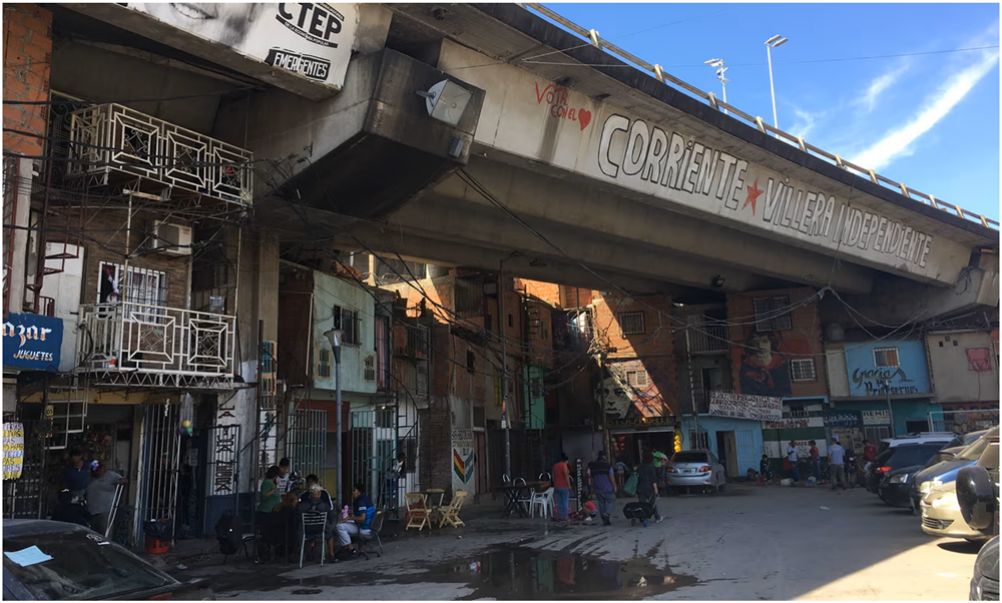
Informal settlement beneath a bridge in Buenos Aires, Argentina. The settlement increases the likelihood of future fire accidents in highway sections that may have previously crossed desolate areas. The absence of proper urban planning and design raises challenges for fire brigade accessibility in the event of a fire emergency (Photo by Luisa Rollenhagen).

The changing built environment includes the rapid global growth of informal settlements, particularly in low and middle-income countries^113^. In such contexts, significant transport infrastructure deficits also point towards an upcoming boom in road construction to support regional and cross-border integration for economic prosperity (e.g., ref. ^114^). Beyond the complexities of informal settlement fires per se (e.g., ref. ^115^), this ongoing expansion has implications for other infrastructure fire safety, including bridges (see Fig. 6). First, the not-uncommon proximity of informal settlements to bridge structures (either existing or new) increases the probability of fires spreading from the settlements to bridges (and vice versa). Second, informal settlements often lack formal (risk-based) urban planning and design (e.g., refs. ^116,117^), as well as reliable water supply, posing challenges for emergency services to access and control fires promptly. Consequently, bridges and highway sections that once crossed empty lands may suddenly face the threat of fires with uncertain (i.e., scarcely quantifiable) yet potentially disastrous intensity.

**Fig. 7:**
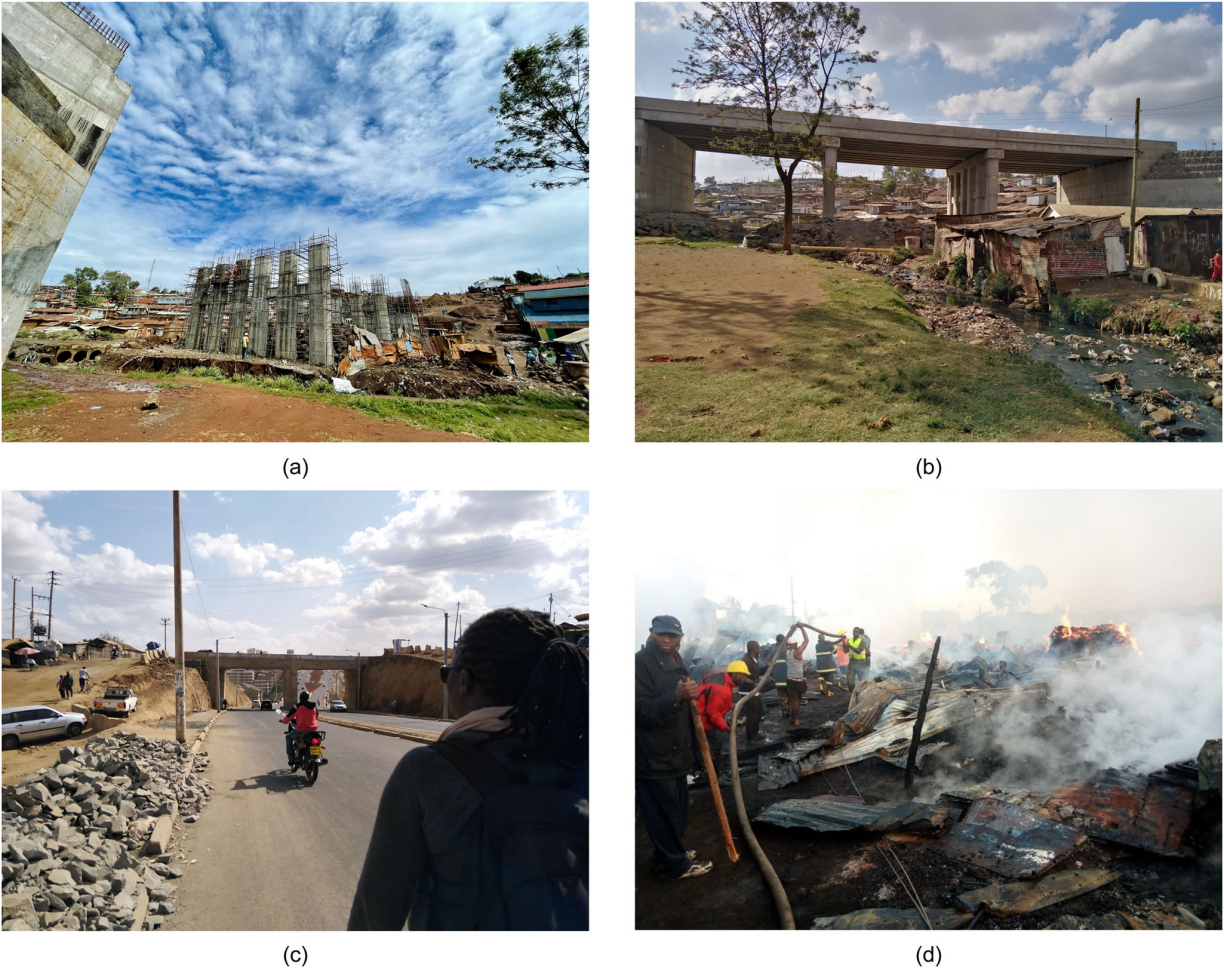
Fire risk characteristics of Missing Link bridge infrastructure through the Kibera settlement in Nairobi, Kenya. **a** Construction of the Missing Link bridge in 2021 (Photo by Joe Mulligan, KDI). **b** Completed Missing Link bridge infrastructure through the Kibera settlement in 2023 (Photo by Joe Mulligan, KDI). **c** Blockages, informal transportation usage, and unfinished surfaces limit access to the bridge and the settlement in 2024 (Photo by Amos Wandera, KDI). **d** Community response to a fire in Laini Saba, Kibera (Photo by Pascal Kipkemboi, KDI).

The Missing Link Road project in Nairobi, Kenya, illustrates some of the mentioned challenges. Residents in Nairobi’s informal settlements often experience fire outbreaks, yielding massive loss of lives, property, and other household assets^118^. As shown in Fig. 7, the project included a bridge that traverses Kibera, Nairobi’s largest informal settlement^119^. Nevertheless, this structure was designed and constructed without considering its connectivity to the settlement^120^, compromising the fire safety of both. Moreover, the lack of a complete ramp or utility infrastructure and the proliferation of informal road uses (see Fig. 7c) limit emergency vehicle accessibility. These challenges are widespread among many other cities currently investing in new transport infrastructures and witnessing a growing informal settlement population.

The long service life of bridges (typically 100 years or more) also exposes them to climate change’s effects (e.g., ref. ^121^). Among the various impacts of climate change on bridge safety (e.g., ref. ^122^), the following aspects directly affect their fire performance: (i) accelerated degradation of construction materials, which intensifies the phenomena discussed above; (ii) increased demand on deformation capacity at free ends, with the potential development of thermal stresses due to the contact with adjacent abutments or spans: in the case of a fire, these stresses are critical in determining bridge performance (e.g., refs. ^44,123^); (iii) higher chance of vehicle-pier collision (with potential fire ignition) due to reduced visibility during fogs and more slippery roads; (iv) an increase in intensity and/or frequency of wildfires; (v) increased intensity/frequency of other hazards (e.g., floods and other hydrometeorological hazards^124,125^) that can cause damage accumulation before the fire event.

Due to uncertain changes in the built environment and long-term multi-hazard interactions, investigating and developing analytical tools for the time-varying reliability and risk of bridges under fire exposure is necessary. To our knowledge, no studies on this subject exist in the literature. These analyses should then be incorporated into multi-hazard lifecycle consequence (e.g., refs. ^82,126^) and resilience (e.g., refs. ^127,128^) calculation methodologies.

## Conclusions

Recent bridge fires highlight the potential to enhance the design of future infrastructures and rebuild failed ones through a commitment to *Design Better* and *Build Back Better*, fostering sustainable development and societal resilience. We first recognize that fires are outputs of the design process. Thus, bounding them through worst-case scenarios inappropriately eliminates one variable from the design optimization process. To cope with this limitation, we propose using hazard-centered design methodologies, which focus on controlling fire dynamics rather than merely bolstering structural strength. The active management of the variables that affect the dynamics of fire allows framing structural fire safety within holistic, multicriteria, and multi-hazard approaches. These approaches enable flexibility in selecting design variables and, consequently, a more optimized design. The forward-looking perspective proposed here not only strives to mitigate the negative impact of fires on bridges but also allows considerations of future risks, deterioration, and the impacts of climate change within the context of a dynamically evolving built environment to be incorporated.

While the potential benefits of this overall approach are promising and substantial, the undeniable and tangible cost of inaction is evident in the ongoing losses inflicted upon society by fires interacting with bridges. Table 1 summarizes our main points of concern and corresponding recommendations. On a final note, we observe that societies have limited resources and their allocation for infrastructure resilience should be based on a comprehensive cost-benefit analysis at a societal scale to effectively prioritize investments. Nevertheless, the methods, approaches, and practical recommendations outlined in this Perspective can enhance decision-making and improve the multidimensional resilience of our built environment.

**Table 1:** Identified limitations in the fire safety design of bridges and suggested actions

Limitation	Recommendations
Fire safety design of bridges is often overlooked.	• Bridge design codes should require fire consequence analysis. • The lack of regulations provides an opportunity to *Design Better* future infrastructures and *Build Back Better* damaged ones.
The conventional (prescriptive) definition of **“**fire resistance**”** does not capture actual heating conditions, limits design solutions to tested options, ignores a bridge**’**s capacity to retain integrity despite localized failures and could be unconservative.	• Implement performance-based design and assessment approaches. • Define explicit performance objectives considering a spectrum of damage states. • Clearly distinguish between deterministic damage, reliability, vulnerability, risk, functionality, and resilience performance objectives. • Adopt fire models that capture combustion process features, fire dynamics, and fire-structure interaction. • Analyze the thermo-mechanical response of bridges rather than adopting implicit temperature-based failure criteria.
Fire scenarios are treated as inputs for design.	• Exploit the fire-structure interaction to treat fire scenarios (and, therefore, fire intensity) as design variables. • Optimize the balance between increasing structural capacity and decreasing fire intensity (rather than simply boosting structural capacity) to bound maximum fire consequences within a selected threshold. • Exploit the inherent fire capacity of bridges (in place of active and passive fire protection).
If fire hazards are considered, they are incorporated as an add-on to a design that has been optimized for other ordinary and extreme loads.	• Implement a holistic engineering approach that integrates adequate design and assessment methods for all relevant hazards, including fire, from the early design stages. • Integrate fire safety into multi-hazard design and assessment frameworks, accounting for interactions and asynergies while exploiting synergies. • Multi-hazard design also enhances environmental, economic, and social sustainability.
Fire safety design and assessment typically assume static representations of the built environment.	• Leverage future-oriented risk modeling frameworks that capture the impacts of new technologies, expanding/densifying cities, informal settlement growth, aging and weathering, and climate change on fire safety. • Develop analytical tools for the time-varying reliability and risk of bridges under fire exposure.
